# Association between risk of preeclampsia and maternal plasma trimethylamine-N-oxide in second trimester and at the time of delivery

**DOI:** 10.1186/s12884-020-02997-7

**Published:** 2020-05-19

**Authors:** Xin Huang, Zuodong Li, Zhou Gao, Dapeng Wang, Xiaohui Li, Ying Li, Chunmei Mi, Jun Lei

**Affiliations:** 1 Department of Epidemiology, Hunan Normal University School of Medicine, 371 Tongzipo Road, Yuelu District, Changsha, 410013 Hunan, Province China; 2 Department of Obstetrics and Gynecology, The Third Xiangya Hospital of Central South University, Changsha, 410013 Hunan China; 3 Key Laboratory of Environmental Pollution Monitoring and Disease Control, Guizhou Medical University, Guiyang, 550025 Guizhou China; 4 Hunan Key Laboratory for Bioanalysis of Complex Matrix Samples, Changsha, 411000 Hunan China; 5 Department of Pharmacology, Central South University School of Pharmaceutical Sciences, Changsha, 410013 Hunan China; 6 Department of Health Management, The Third Xiangya Hospital of Central South University, Changsha, 410013 Hunan China

**Keywords:** Trimethylamine-N-oxide, Preeclampsia, Early onset preeclampsia, Sever preeclampsia, Nested case-control study

## Abstract

**Background:**

The data on the association between the microbiota-dependent metabolite trimethylamine-N-oxide (TMAO) during pregnancy and risk of preeclampsia (PE) is limited.

**Methods:**

We, therefore, conducted a prospective nested case control study during Sep 2017 to Dec 2018 to examine the association between plasma TMAO measured during pregnancy and the risk of PE. Total of 17 patients diagnosed with early onset PE (EOPE), 49 with late onset PE (LOPE) and 198 healthy controls were enrolled. Blood samples were collected at 15–23 weeks gestation and time at delivery. The Logistic regression model was used to assess the odds ratio (OR) and 95% confidence interval (CI) for TMAO and risk of PE, EOPE, LOPE, mild PE, and severe PE.

**Results:**

We found that the mean TMAO levels of overall subjects in the second trimester (T2) and at the time of delivery (TD) were 90.39 μg/m^3^ (standard deviation (SD) =45.91) and 175.01 μg/m^3^ (SD = 160.97), respectively. No significant spearman correlation was found between the TMAO in those two periods (*p* > 0.05). T2 TMAO was not significantly associated with risk of PE or risk of any PE subtypes (*p* > 0.05). However, TD TMAO was significant associated with risk of PE, EOPE and severe PE (adjusted OR and 95%CI were 1.24(1.09, 1.40), 1.62(1.29, 2.03), and 1.41(1.17, 1.70)) per 50 μg/m^3^ increment, respectively).

**Conclusion:**

Our study found that plasma TMAO level would alter over the course of pregnancy. The major role of TMAO in PE development might be in the accelerating process not in the initiation.

## Background

Preeclampsia (PE) complicates 2–5% of pregnancies and is a major cause of maternal, fetal, and neonatal morbidity and mortality [1]. Recent researchers found that the intestinal microbiota plays an important role in the development of PE [2–4]. However, it is still unclear how intestinal microbiota participates in the pathogenesis of PE.

Microbes that live in the gut could produce a vast number of metabolites, many of which are dependent on the diet of the host [5] and absorbed into the circulation, chemically modified by the host, and eventually exert their functions in host physiology and pathophysiology [6]. Food containing phosphatidylcholine, choline, and carnitine, can be used by gut microbes as a carbon fuel source and high levels of trimethylamine (TMA) would be metabolized at the same time [7, 8]. TMAs which are carried via the portal circulation to the liver, are oxidized further into TMA-N-oxide (TMAO) by a family of enzymes, flavin monooxygenases (FMOs) [7, 8]. Plasma TMAO levels have been associated with risk of atherosclerotic cardiovascular disease (ASCVD) in multiple distinct clinical studies [8–11]. The underlying mechanisms might involve increased coronary atherosclerotic plaque burden [8], accelerated endothelial dysfunction [9], and risk for thrombotic events [11] induced by TMAO in human subjects.

PE shared similar mechanisms with ASCVD whereby both of them involved oxidative stress and endothelial dysfunction [12–15]. They have common risk factors such as obesity, diabetes, and hypertension [13–15]. Recently, a study conducted on the reduced uterine perfusion pressure (RUPP) rat model implies that TMAO was also involved in the development of PE [16]. However, the role of TMAO in PE development as an initiator or a trigger could not be answered by RUPP model. Moreover, the data on human beings is still lacking. We, therefore, conducted a prospective nested case control study in Changsha, China, to examine the association between plasma TMAO measured during pregnancy and the risk of PE.

## Methods

### Study population

We enrolled subjects from an ongoing prospective cohort which aimed at maternal depression screening and associated risk factors. The study was performed at the Third Xiangya Hospital of Central South University (TXYHCSU) from September 2017 until December 2020. The enrollment criteria for the cohort were singleton pregnancy, ≥20 years old, with no history of mental disease. All qualified pregnant women at 12–14 weeks gestation were recruited when they attended first prenatal visit and would be followed till 6 weeks after delivery. In the present nested case-control study, all patients in the cohort diagnosed with PE and have delivered before the end of 2018, with no history of chronic diabetes mellitus, renal disease, or other chronic cardiovascular disease were recruited in case group. We matched each PE case with 3 controls who were normotensive singleton pregnant women, without chronic diseases, with similar age (within ±3 years), and blood samples were collected. Finally, 17 patients diagnosed with early onset PE (EOPE), 49 with late onset PE (LOPE) and 198 healthy controls were enrolled.

### Data collection and diagnosis criteria

The study protocol were reviewed and approved by the Ethical and Confidentiality Committee of Central South University. Written informed consent was obtained from all participants. A face-to-face interview was conducted at baseline using a structured questionnaire to collect demographic, environmental and medical information. Enrolled subjects were followed at the following 5 time points: 15–23 weeks gestation (second trimester, T2), 24–32 weeks gestation, time at delivery (TD), 2–4 weeks and 6 weeks postpartum. At T2 and TD, 5 ml fasting blood samples were collected in tubes with heparin as anticoagulant and centrifuged at 1620 g at 4 °C for 5 min. Then, the plasma was detached and stored at − 80 °C for subsequent analysis of TMAO. Information on maternal complications was abstracted from medical charts. PE was diagnosed as de novo hypertension (blood pressure, BP ≥140/90 mmHg) after 20 weeks gestation accompanied with proteinuria (≥ 1+ on dipstick). PE was further classified as mild PE (BP ≥140/90 mmHg and < 160/110 mmHg, proteinuria ≥1+ and < 2+ on dipstick, and without symptoms of severity), severe PE (BP ≥160/110 mmHg, or proteinuria ≥2+ on dipstick, or with symptoms of severity such as thrombocytopenia, impaired liver function, de novo renal insufficiency, pulmonary edema, cerebral or visual disturbance), EOPE (diagnosed before 34 weeks gestation), or LOPE (diagnosed at or after 34 weeks gestation).

### Plasma TMAO concentration assays

We measured TMAO concentrations in Hunan Key Laboratory for Bioanalysis of Complex Matrix Samples using ultrahigh performance liquid chromatography-tandem mass spectrometry (UHPLC-MS/MS) as described by Awwad HM et al. [17] and the details was described in the supplementary (Additional file 1). In brief, all the samples were kept in the auto-sampler at 10 °C TMAO and d9-TMAO were monitored in positive-ion mode with multiple reaction monitoring of precursor and characteristic production transitions of m/z 76.3 → 58.4 and 85.1 → 66.3, respectively. Various concentrations of non-isotopically labeled TMAO were mixed with a fixed amount of internal standard d9-TMAO to prepare the calibration curves for quantification of plasma TMAO. For quality assurance, 8 different quality-control samples with TMAO concentrations ranging between 2 and 500 ng/mL were used for the evaluation of accuracy and precision. Samples with TMAO concentration exceeding 500 ng/mL were diluted and the final concentrations were calculated with use of appropriate dilution factor. The accuracy of quality-control samples was within the range of 85–105% of the nominal values, the intra- and inter assay coefficient of variations (CVs) were all below 6%, and the absolute recovery was between 85 and 106%. All of the assays were performed without knowledge of PE status.

### Statistical analysis

The distribution of maternal characteristics was compared between PE and control groups by Chi-squared tests. Prior to describe the distribution of TMAO concentration, the normality assumption of residuals was tested using Shapiro-Wilk test. However, the significant departures from normality were observed (*p* < 0.05). Median and range were used to describe the distribution of TMAO among overall subjects. Individual change of TMAO was TD TMAO minus the T2 TMAO. The spearman correlation between the change of plasma TMAO and TMAO in T2 or TD was further estimated. Unconditional logistic regression model was used to calculate odds ratios (OR) and 95% confidence intervals (CI) for the associations between TMAO and risk of PE, EOPE, LOPE, mild PE, and severe PE, by different times. The exposure of TMAO was evaluated in two ways: 1) categorized by quartiles; 2) treated as a continuous variable, and results were presented for the change in health outcome per 50 μg/m^3^. Potential confounding variables included in multivariate models were year of education (< 12, ≥12), active and/or passive smoking during pregnancy (yes or no), pre-pregnancy body mass index (BMI, < 18.5, 18.5–22.9, and ≥ 23 kg/m^2^), and fetal gender (male or female). Statistical significance was all assessed at the two-tailed 0.05 level. All analyses were performed using SAS, version 9.2 (SAS Institute, Inc., Cary, NC).

## Results

### General characteristics

Compared to controls, women with PE were more likely to be less educated (*p* = 0.040). There were no significant differences in the distribution of pre-pregnancy BMI, exposure to smoking during pregnancy and fetal gender between preeclampsia and controls (Table 1).

**Table 1:** Distributions of characteristics between preeclampsia and control groups

Characteristics	PE (*N* = 66)	Control (*N* = 198)	*p*
Year of education, years			
≤ 12	54 (81.80)	136 (68.70)	0.040
> 12	12 (18.20)	62 (31.30)	
Pre-pregnancy BMI (Kg/m^2^)			
< 18.5	12 (18.18)	32 (16.16)	0.842
18.5–22.9	38 (57.58)	122 (61.62)	
≥ 23	16 (24.24)	44 (22.22)	
Active or passive smoking during pregnancy			
Yes	19 (28.78)	40 (20.20)	0.363
No	48 (70.75)	158 (79.80)	
Fetal gender			
Male	38 (57.58)	100 (50.51)	0.319
Female	28 (42.42)	98 (49.49)	

Abbreviation: *PE* preeclampsia; *BMI* body mass index

### Distribution of maternal plasma TMAO

Table 2 presented distribution of maternal plasma TMAO concentration in T2 and TD for the overall study subjects. The median TMAO concentration for T2 and TD were 84.80 μg/m^3^ and 138.60 μg/m^3^, respectively. More than 75% subjects showed increased TMAO concentration as they progressed from T2 to TD (Table 2). However, no significant spearman correlation was found between the plasma TMAO in those two periods (*p* = 0.157, Table 3). Direct comparison on TMAO between control and PE was listed in the supplementary (Table S1) and the results were similar to the following logistic regression analysis (Tables 4 and 5).

**Table 2:** Maternal plasma TMAO concentration during pregnancy among overall subjects(*N* = 264)*

Characteristic	T2 plasma TMAO (μg/m^3^)	TD plasma TMAO (μg/m^3^)	Change of TMAO (μg/m^3^)
Mean ± SD	90.39 ± 45.91	175.01 ± 160.97	84.62 ± 159.32
Median	84.80	138.60	54.13
Minimum	4.15	21.0	− 146.02
Maximum	305.00	1560.00	1255.00
First Quartile	57.58	86.30	0.00
Third Quartile	116.36	207.04	123.04
Spearman Correlation with T2 plasma TMAO	1.00	0.09^*^	−0.37ǂ
Spearman Correlation with TD plasma TMAO	0.09	1.00	0.87ǂ

^*^p = 0.157; ǂ *p* < 0.001

Abbreviation: *TMAO* trimethylamine-N-oxide; *T2* the second trimester; *TD* the time of delivery

**Table 3:** Comparison on maternal plasma TAMO concentration between preeclampsia and control groups

Group	T2 plasma TMAO (median (Q1, Q3), μg/m^3^)	TD plasma TMAO (median (Q1, Q3), μg/m^3^)	Change of TMAO (median (Q1, Q3), μg/m^3^)
Control (*N* = 198)	84.05 (60.44, 113.90)	134.96 (78.10, 202.15)	53.71(− 2.60, 115.52)
PE (*N* = 66)	92.40 (53.80, 126.36)	151.01 (103.45, 280.48)	57.47 (8.32, 211.50)
Difference with control (95%CI)^a^	7.45(− 6.92, 21.83)	33.25 (8.54, 57.97)	24.36(−9.11, 57.84)
*p*^b^	0.296	0.010	0.167
EOPE (*N* = 17)	72.30 (54.58, 109.66)	370.00 (132.37, 514.86)	297.70 (34.63, 454.05)
Difference with control (95%CI)^a^	−2.62(−23.44, 18.19)	191.25 (52.33,330.16)	196.49 (63.67, 329.31)
*p*^b^	0.791	< 0.001	< 0.001
LOPE (*N* = 49)	93.30(53.80, 149.00)	138.71 (101.66, 207.00)	30.63 (0.00, 122.42)
Difference with control (95%CI)^a^	12.33(−5.03, 29.70)	14.55(−11.16, 40.28)	−4.66(−37.17, 27.84)
*p*^b^	0.162	0.245	0.766
Mild PE (*N* = 41)	103.45 (53.80, 149.00)	138.71 (100.68, 207.08)	34.63(−1.03, 130.69)
Difference with control (95%CI)^a^	13.71(−6.17, 33.60)	15.00(−13.00, 43.02)	−3.70(−40.44, 33.04)
*p*^b^	0.170	0.275	0.819
Sever PE (*N* = 25)	85.10 (54.60, 109.66)	168.24 (128.14, 476.65)	85.23 (27.96, 340.35)
Difference with control (95%CI)^a^	0.15(−17.69, 17.99)	78.93 (26.88, 130.98)	88.10 (21.03, 155.17)
*p*^b^	0.976	0.002	0.006

^a^ 95% CIs were estimated with the Hodges-Lehmann method

^b^ Based on Wilcoxon rank-sum test

Abbreviation: *TMAO* trimethylamine-N-oxide; *PE* preeclampsia; *EOPE* early onset preeclampsia; *LOPE* late onset preeclampsia; *T2* the second trimester; *TD* the time of delivery; *Q1* first quartile; *Q3* third quartile; *CI* confident interval

**Table 4:** Association between preeclampsia and maternal plasma TAMO concentration in the second trimester

PE type	Quartile for TMAO concentration in the second trimester (μg/m^3^)				*p* for trend	Per 50 μg/m^3^ increment
	Q1 (< 57.58)	Q2 (57.58- < 84.80)	Q3 (84.80- < 116.36)	Q4 (≥116.36)		
PE						
Case / Control	21/ 45	9/ 57	14/ 52	22/ 44		
OR (95%CI)	Reference	0.34 (0.14, 0.81)	0.58 (0.26, 1.27)	1.07 (0.52, 2.22)	0.611	1.33 (0.98, 1.79)
OR^a^_adj_ (95%CI)	Reference	0.30 (0.12, 0.75)	0.57 (0.24, 1.32)	1.00 (0.47, 2.14)	0.867	1.23 (0.90, 1.68)
EOPE						
Case / Control	6/ 45	3/ 57	4/ 52	4/ 44		
OR (95%CI)	Reference	0.40 (0.09, 1.67)	0.58 (0.15, 2.17)	0.68 (0.18, 2.58)	0.643	0.89 (0.47, 1.67)
OR^a^_adj_ (95%CI)	Reference	0.34 (0.08, 1.53)	0.52 (0.13, 2.18)	0.66 (0.16, 2.65)	0.372	0.75 (0.39, 1.44)
LOPE						
Case / Control	15/ 45	6/ 57	10/ 52	18/ 44		
OR (95%CI)	Reference	0.32 (0.11, 0.88)	0.58 (0.24, 1.41)	1.23 (0.55, 2.74)	0.389	1.48 (1.06, 2.05)
OR^a^_adj_ (95%CI)	Reference	0.40 (0.16, 1.00)	0.61 (0.24, 1.56)	1.12 (0.48, 2.59)	0.521	1.41 (1.00, 1.98)
Mild PE						
Case / Control	14/ 45	4/ 57	7/ 52	16/ 44		
OR (95%CI)	Reference	0.23 (0.07, 0.73)	0.43 (0.16, 1.17)	1.17 (0.51, 2.68)	0.497	1.55 (1.09, 2.20)
OR^a^_adj_ (95%CI)	Reference	0.25 (0.05, 1.19)	0.43 (0.15, 1.30)	1.19 (0.50, 2.86)	0.510	1.35 (0.69, 2.64)
Severe PE						
Case / Control	7/ 45	5/ 57	7/ 52	6/ 44		
OR (95%CI)	Reference	0.56 (0.17, 1.90)	0.87 (0.28, 2.66)	0.88 (0.27, 2.82)	0.999	0.98 (0.58, 1.66)
OR^a^_adj_ (95%CI)	Reference	0.53 (0.15, 1.87)	0.89 (0.26, 2.97)	0.71 (0.20, 2.50)	0.550	0.83 (0.48, 1.43)

a: Adjusted for year of education, pre-pregnancy BMI, smoking during pregnancy, fetal gender;

Abbreviation: *TMAO* trimethylamine-N-oxide; *PE* preeclampsia; *EOPE* early onset preeclampsia; *LOPE* late onset preeclampsia; *OR* odds ratio; *CI* confidence interval

**Table 5:** Associations between preeclampsia and maternal plasma TMAO concentration at the time of delivery

PE type	Quartile for TMAO concentration in the third trimester (μg/m^3^)				*p* for trend	Per 50 μg/m^3^ increment
	Q1 (< 86.30)	Q2 (86.30- < 138.60)	Q3 (138.60- < 207.04)	Q4 (≥207.04)		
PE						
Case / Control	8/ 57	22/ 45	14/ 52	22/ 44		
OR (95%CI)	Reference	3.48 (1.42, 8.56)	1.92 (0.74, 4.94)	3.56 (1.45, 8.76)	0.033	1.23 (1.09, 1.37)
OR^a^_adj_ (95%CI)	Reference	3.62 (1.39, 9.43)	2.10 (0.80, 5.55)	4.25 (1.62, 11.10)	0.018	1.24 (1.09, 1.40)
EOPE						
Case / Control	1/ 57	5/ 45	1/ 52	10/ 44		
OR (95%CI)	Reference	6.33 (0.71, 56.16)	1.10 (0.07, 17.98)	12.96 (1.60, 105.04)	0.008	1.59 (1.29, 1.96)
OR^a^_adj_ (95%CI)	Reference	5.76 (0.64, 52.27)	1.15 (0.09, 14.88)	14.86 (1.66, 129.85)	0.010	1.62 (1.29, 2.03)
LOPE						
Case / Control	7/ 57	17/ 45	13/ 52	12/ 44		
OR (95%CI)	Reference	3.08 (1.17, 8.06)	2.04 (0.75, 5.49)	2.22 (0.81, 6.11)	0.273	1.12 (0.99, 1.27)
OR^a^_adj_ (95%CI)	Reference	3.41 (1.21, 9.56)	2.23 (0.80, 6.22)	2.70 (0.93, 7.85)	0.190	1.12 (0.98, 1.28)
Mild PE						
Case / Control	7/ 57	13/ 45	10/ 52	11/ 44		
OR (95%CI)	Reference	2.35 (0.87, 6.39)	1.57 (0.56, 4.41)	2.04 (0.73, 5.68)	0.319	1.14 (1.00, 1.31)
OR^a^_adj_ (95%CI)	Reference	2.16 (0.75, 6.24)	1.83 (0.62, 5.39)	2.61 (0.87, 7.83)	0.184	1.16 (1.00, 1.35)
Severe PE						
Case / Control	1/ 57	9/ 45	4/ 52	11/ 44		
OR (95%CI)	Reference	11.39 (1.39, 93.27)	4.39 (0.48, 40.50)	14.25 (1.77, 114.59)	0.015	1.40 (1.18, 1.67)
OR^a^_adj_ (95%CI)	Reference	12.25 (1.41, 106.65)	4.42 (0.48, 40.92)	14.37 (1.74, 118.77)	0.019	1.41 (1.17, 1.70)

a Adjusted for year of education, pre-pregnancy BMI, smoking during pregnancy, fetal gender;

Abbreviation: *TMAO* trimethylamine-N-oxide; *PE* preeclampsia; *EOPE* early onset preeclampsia; *LOPE* late onset preeclampsia; *OR* odds ratio; *CI* confidence interval

### Association between plasma TMAO and PE

With adjustment for education, smoking during pregnancy, pre-pregnancy BMI, and fetal gender, T2 TMAO was not significantly associated with risk of PE or risk of any PE subtypes (*p*-trend > 0.05, Table 4). However, TD TMAO was significant positively associated with risk of PE, EOPE and severe PE (adjusted OR and 95%CI were 1.24(1.09, 1.40), 1.62(1.29, 2.03), and 1.41(1.17, 1.70)) per 50 μg/m^3^ increment, respectively) (Table 5). Comparing the highest TMAO exposure with the lowest quartile, the adjusted ORs for PE were 4.25 (95% CI: 1.62, 11.10, *p*-trend = 0.018); for EOPE 14.86 (95% CI: 1.66, 129.85, *p*-trend = 0.010); and for severe PE were 14.37 (95% CI: 1.74, 118.77, *p*-trend = 0.019).

## Discussion

### Main findings

Our nested case-control study found that the maternal plasma TMAO would alter over the course of pregnancy. Maternal plasma TMAO in second trimester was not associated with PE. However, the plasma TMAO level among PE, EOPE and severe PE patients was significantly increased at the time of delivery.

### Interpretation

Circulating TMAO is formed via a two-step metaorganismal pathway [7, 8]. Specifically, following food intake, gut microbes form TMA, and then host hepatic FMOs catalyze the conversion of TMA into TMAO [7, 8]. Interventions involving major gut microbiota or dietary alterations would lead to TMAO alterations, such as antibiotics, diet intervention, and bariatric surgery [18–21]. Our findings suggested that TMAO level would also alter during natural pregnancy. To the best of our knowledge, our work is the first longitudinal study to investigate TMAO levels within individual pregnant women from T2 to TD. Koren et al. [22] had followed 91 pregnant women and found that the gut microbial community composition was profoundly altered over the course of pregnancy. Though food consumption pattern does not change significantly throughout the pregnancy period, women were more likely tended to consume more red meat and eggs during pregnancy [23]. More than 75% subjects showed increased TMAO as T2 progressing to TD and the degree of alteration was not related to the T2 TMAO level. Thus, we speculated that altered gut microbiota components, plus increased red meat and eggs consumption during pregnancy might contribute to the observed TMAO alteration in our study. However, we do not have any data about food consumption or gut microbiota variation occurring in the study subjects during the progress of pregnancy, the underlying mechanisms need further validation.

Plasma TMAO levels have been observed associated with risk of ASCVD [12–15], kidney failure [24], cancer [25] and gestational diabetes mellitus [26] in clinical studies. Recently, a study conducted on the RUPP rat model implies that TMAO was also involved in the development of PE [16]. The findings in the present study supported the finding that PE patients had elevated TD TMAO. However, elevated TMAO in T2 was no associated with PE. And elevated TD TMAO was only found in severe PE but not mild PE patients. The difference in those associations might imply a major role of TMAO in PE progression but not in PE initiation. In consistence with our findings, Wang et al. [8] found exacerbated atherosclerotic lesions after high TMAO exposure which is the major pathogenic events in preeclampsia. Additionally, in preeclampsia, the coagulation and fibrinolytic cascades are highly activated, accompanied by pathological blood rheology and endothelial dysfunction [13]. Studies conducted by Chen et al. [16] and Seldin et al. [27] indicating promoted recruitment of activated leukocytes to endothelial cells by elevated TMAO exposure. In a recent issue of Cell, Zhu et al. [11] also confirmed the role of TMAO in thrombosis was enhancing platelet hyper-reactivity.

LOPE and EOPE are two distinct phenotypes of PE with different mechanisms [28]. Our study confirmed this hypothesis, finding association only existing between maternal plasma TMAO and EOPE. EOPE appears to be more related to placental disorder [29]. However, LOPE seems to be more linked to maternal constitutional factors [28]. We speculated that elevated TMAO exposure might aggravate the aberrant spiral artery remodeling and poor placentation. The specific mechanism requires further research.

### Strengths and limitations

Strengths and limitations should be considered when interpreting the study findings. The nested case control study with prospective design allowed us observing the TMAO variation during pregnancy in the same individual. Information on maternal complications was obtained from medical records, which minimized the potential misclassification of the outcomes. Furthermore, we have data on maternal education, smoking during pregnancy, pre-pregnancy BMI, and fetal gender which allowed adjusting for several important potential confounding factors. However, the limitation of our study should be considered when interpreting the study findings. In present study, we have only accessed the plasma TMAO in second trimester and at the time of delivery. The distribution and alteration of TMAO in other period, such as, preconception, the first trimester and postpartum need further investigation. The TMAO level was associated the various factors including diet pattern, gut microbes, liver function and FMOs genotype [7, 8, 22], however their associations with PE were not discussed at present study. The sample size of our study is small and the replication of our findings in other independent population is needed.

## Conclusion

Maternal plasma TMAO would alter over the course of pregnancy. Maternal plasma TMAO in second trimester was not associated with PE. However, the plasma TMAO level among PE patients was significantly increased at the time of delivery. The major role of TMAO in PE development was in the accelerating process not in the initiation.

## Supplementary information

**Additional file 1.** Methods for Plasma TMAO concentration assay

**Additional file 2 Table S1.** Comparison on maternal plasma TAMO concentration between preeclampsia and control groups

## Data Availability

The datasets used and analyzed during the current study are available from the corresponding author on reasonable request.

## Abbreviation

TMAO: Trimethylamine-N-oxide
PE: Preeclampsia
EOPE: Early onset preeclampsia
LOPE: Late onset preeclampsia
BMI: Body mass index
T2: The second trimester
TD: The time of delviery
