# Supplementary material for: Association between risk of preeclampsia and maternal plasma trimethylamine-N-oxide in second trimester and at the time of delivery
Source: BMC Pregnancy Childbirth. 2020 May 19;20:302. doi: 10.1186/s12884-020-02997-7 (PMC7236207; doi:10.1186/s12884-020-02997-7)
Supplement: Supplementary file 1 — Additional file 1. Methods for Plasma TMAO concentration assay [file 12884_2020_2997_MOESM1_ESM.docx]

**Additional files:**

Additional file 1: Methods for Plasma TMAO concentration assay

Additional file 2: Table S1. Comparison on maternal plasma TAMO concentration between preeclampsia and control groups

**Additional file 1**

**Methods for Plasma TMAO concentration assay**

The frozen plasma were thawed and centrifuged at 5,500×g and 4^°^C for 5 min. Then 100μL plasma samples were added with 300μL internal standard solution which contained a mixture of 10μmol/L of d9-TMAO prepared in methanol/ acetonitrile (15:85) and 0.2% formic acid. The mixture was briefly vortex mixed and centrifuged at 10,000 ×g, 4◦C for 5 min. The supernatant was transferred to another tube and evaporated to dryness using a vacuum drying concentrator at 25^◦^C. Finally, the drying residue was dissolved in100 μL of methanol-acetonitrile (25:75, v: v), followed by vortex-mixed for 2 min, and then centrifuged at 10,000 ×g for 5 min. The corresponding supernatant was transferred to an auto-sampler vial with an insert (LVI, 150μL, Waters) and 20μL was injected into the UHPLC–MS/MS system for analysis. UHPLC–MS/MS analyses were carried out using an UPLC Acquity coupled to a MicroMass Quattro Premier XE tandem quadrupole mass spectrometer (Waters Corporation, Milford, MA, USA). Chromatographic separation was performed using an Acquity UPLC BEH HILIC column (100mm×2.1 mm; 1.7μm Waters Corporation, Milford, MA, USA). The column temperature was set to 30°C, and the flow rate was set as 0.4 mL/min and composed of Water (A) (containing 15mmol/L ammonium formate, pH = 3.5) and acetonitrile (B) as the mobile phase. The condition of isocratic elution was set to 60% B and the total running time was 3 min. All the samples were kept in the auto-sampler at 10^◦^C. TMAO and d9-TMAO were monitored in positive-ion mode with multiple reaction monitoring of precursor and characteristic production transitions of m/z 76.3→58.4 and 85.1→66.3, respectively. Various concentrations of non-isotopically labeled TMAO were mixed with a fixed amount of internal standard d9-TMAO to prepare the calibration curves for quantification of plasma TMAO. For quality assurance, 8 different quality-control samples with TMAO concentrations ranging between 2 and 500ng/mL were used for the evaluation of accuracy and precision. Any sample with a TMAO concentration exceeding 500ng/mL was diluted and the final concentration was calculated with use of appropriate dilution factor. The accuracy of quality-control samples was within the range of 85-105% of the nominal values, the intra- and inter assay coefficient of variations (CVs) were all below 6%, and the absolute recovery was between 85% and 106%. All of the assays were performed without knowledge of PE status.
