# Supplementary material for: Association between risk of preeclampsia and maternal plasma trimethylamine-N-oxide in second trimester and at the time of delivery
Source: BMC Pregnancy Childbirth. 2020 May 19;20:302. doi: 10.1186/s12884-020-02997-7 (PMC7236207; doi:10.1186/s12884-020-02997-7)
Supplement: Supplementary file 2 — Additional file 2 Table S1. Comparison on maternal plasma TAMO concentration between preeclampsia and control groups [file 12884_2020_2997_MOESM2_ESM.docx]

**Additional file 2**

Table S1. Comparison on maternal plasma TAMO concentration between preeclampsia and control groups

| Group | | T2 plasma TMAO  (median (Q1, Q3), µg/m^3^) | TD plasma TMAO (median (Q1, Q3), µg/m^3^) | Change of TMAO  (median (Q1, Q3), µg/m^3^) |
| --- | --- | --- | --- | --- |
| Control (N=198) | | 84.05(60.44, 113.90) | 134.96 (78.10, 202.15) | 53.71( -2.60, 115.52) |
| PE (N=66) | | 92.40(53.80, 126.36) | 151.01(103.45, 280.48) | 57.47 (8.32, 211.50) |
|  | Difference with control ( 95%CI)* | 7.45(-6.92, 21.83) | 33.25(8.54, 57.97) | 24.36(-9.11, 57.84) |
|  | *p*† | 0.296 | 0.010 | 0.167 |
| EOPE (N=17) | | 72.30(54.58, 109.66) | 370.00(132.37, 514.86) | 297.70(34.63, 454.05) |
|  | Difference with control ( 95%CI)* | -2.62( -23.44, 18.19) | 191.25(52.33,330.16) | 196.49(63.67, 329.31) |
|  | *p*† | 0.791 | <0.001 | <0.001 |
| LOPE (N=49) | | 93.30( 53.80, 149.00) | 138.71(101.66, 207.00) | 30.63(0.00, 122.42) |
|  | Difference with control ( 95%CI)* | 12.33(-5.03, 29.70) | 14.55( -11.16, 40.28) | -4.66(-37.17, 27.84) |
|  | *p*† | 0.162 | 0.245 | 0.766 |
| Mild PE (N=41) | | 103.45(53.80, 149.00) | 138.71(100.68, 207.08) | 34.63(-1.03, 130.69) |
|  | Difference with control ( 95%CI)* | 13.71(-6.17, 33.60) | 15.00(-13.00, 43.02) | -3.70(-40.44, 33.04) |
|  | *p*† | 0.170 | 0.275 | 0.819 |
| Sever PE (N=25) | | 85.10(54.60, 109.66) | 168.24(128.14, 476.65) | 85.23(27.96, 340.35) |
|  | Difference with control ( 95%CI)* | 0.15(-17.69, 17.99) | 78.93(26.88, 130.98) | 88.10(21.03, 155.17) |
|  | *p*† | 0.976 | 0.002 | 0.006 |

* 95% CIs were estimated with the Hodges-Lehmann method.

† Based on Wilcoxon rank-sum test.

Abbreviation: TMAO, trimethylamine-N-oxide; PE, preeclampsia; EOPE, early onset preeclampsia; LOPE, late onset preeclampsia; T2, the second trimester; TD, the time of delivery; Q1, first quartile; Q3, third quartile; CI, confident interval
